# OzButterflies – a high quality open database of multispectral images and spectra of tropical to temperate Australian butterflies

**DOI:** 10.1093/database/baag040

**Published:** 2026-07-24

**Authors:** Marilia Fernandes Erickson, Hansani S S Daluwatta Galappaththige, Donald James Mclean, Diogo Jackson de Aquino Silva, Mariel Fulham, Zoe Wild, Michael B J Kelly, Christopher J Irving, Daniele Carlesso, Georgina E Binns, Sanni A Silvasti, Liisa Hämäläinen, Louis G O’Neill, Kiara L’Herpiniere, Johanna Mappes, David W Kikuchi, Hannah M Rowland, Michelle Power, Thomas E White, Darrell J Kemp, Marie E Herberstein

**Affiliations:** School of Natural Sciences, Faculty of Science and Engineering, Macquarie University, Sydney, NSW 2109, Australia; School of Natural Sciences, Faculty of Science and Engineering, Macquarie University, Sydney, NSW 2109, Australia; School of Natural Sciences, Faculty of Science and Engineering, Macquarie University, Sydney, NSW 2109, Australia; Department of Physiology and Behavior, Universidade Federal do Rio Grande do Norte, Natal, RN 59078-900, Brazil; School of Life and Environmental Sciences, The University of Sydney, Camperdown, NSW, 2006, Australia; School of Natural Sciences, Faculty of Science and Engineering, Macquarie University, Sydney, NSW 2109, Australia; Centre for the Advanced Study of Collective Behaviour, University of Konstanz, 78464 Konstanz, Germany; Department of Biology, University of Konstanz, 78464 Konstanz, Germany; School of Natural Sciences, Faculty of Science and Engineering, Macquarie University, Sydney, NSW 2109, Australia; Evolutionary Biomechanics, Zoological Institute and Museum, University of Greifswald, Loitzer Str. 26, 17489, Greifswald, Germany; School of Natural Sciences, Faculty of Science and Engineering, Macquarie University, Sydney, NSW 2109, Australia; Department of Biological and Environmental Science, University of Jyväskylä, Jyväskylä 40014, Finland; Centre for the Advanced Study of Collective Behaviour, University of Konstanz, 78464 Konstanz, Germany; Department of Biology, University of Konstanz, 78464 Konstanz, Germany; Department of Collective Behavior, Max Planck Institute of Animal Behavior, 78464 Konstanz, Germany; School of Natural Sciences, Faculty of Science and Engineering, Macquarie University, Sydney, NSW 2109, Australia; School of Natural Sciences, Faculty of Science and Engineering, Macquarie University, Sydney, NSW 2109, Australia; School of Natural Sciences, Faculty of Science and Engineering, Macquarie University, Sydney, NSW 2109, Australia; Department of Biological and Environmental Science, University of Jyväskylä, Jyväskylä 40014, Finland; School of Natural Sciences, Faculty of Science and Engineering, Macquarie University, Sydney, NSW 2109, Australia; Australian Wildlife Conservancy, Subiaco East, Western Australia 6008, Australia; School of Natural Sciences, Faculty of Science and Engineering, Macquarie University, Sydney, NSW 2109, Australia; Organismal and Evolutionary Biology Research Programme, Faculty of Biological and Environmental Sciences, University of Helsinki, Viikinkaari 1, 00790 Helsinki; Department of Integrative Biology, Oregon State University, Corvallis, OR 97331, United States; Department of Evolution, Ecology and Behaviour, Institute of Infection, Veterinary and Ecological Sciences, University of Liverpool, L69 7ZB, UK; School of Natural Sciences, Faculty of Science and Engineering, Macquarie University, Sydney, NSW 2109, Australia; School of Life and Environmental Sciences, The University of Sydney, Camperdown, NSW, 2006, Australia; School of Natural Sciences, Faculty of Science and Engineering, Macquarie University, Sydney, NSW 2109, Australia; School of Natural Sciences, Faculty of Science and Engineering, Macquarie University, Sydney, NSW 2109, Australia; Centre for Taxonomy and Morphology, Leibniz Institute for the Analysis of Biodiversity Change, Hamburg 53113, Germany; Department of Biology, University of Hamburg, Hamburg 20148, Germany

## Abstract

Butterflies have been a model system for studying the evolution of colour. This is partly due to their complex patterns that reflect human-visible (VIS) and ultraviolet (UV) light, which are perceived by conspecifics and predators. Many studies have sourced data from publicly available images, but most of these images only consider the visible spectrum of light. Including the UV spectrum is crucial for fully understanding the evolution of butterfly morphology and behavioural ecology. Here we provide standardized images (VIS and UV) of over 4 000 individuals from 16 communities of Australian butterflies. These communities represent different climates and urbanization levels spanning over 2 500 km. The dataset contains at least one individual of 125 different species from five families, constituting over one quarter of Australian butterfly diversity. In addition to photographs, we provide spectral measurements of butterfly wings for at least one individual of each species and sex, and Cytochrome Oxidase subunit 1 (CO1) sequences of 1 635 individuals. All these data are accessible in Zenodo and an associated R package simplifies the download of subsets of the database. This database will be of use to evolutionary biologists and ecologists interested in a broad range of topics related to phenotypic variation.

## Introduction

The formal study of butterflies goes back to at least the 18^th^ century with the description of butterfly metamorphosis by Maria Sibylla Merian [1]. The colours of butterflies particularly fascinated 19^th^ century natural historians such as Darwin, Wallace, Poulton, Bates, and Müller [2–6]. The incredible diversity, variation, convergence, and dimorphism of butterfly colours have contributed significantly to the development of evolutionary theory, particularly in relation to sexual selection [7] and warning signal mimicry [2, 4, 6, 8–10]. Butterfly eye physiology and colour perception have also been intensively studied [11,12]. Recently, butterflies in the *Heliconius* and *Bicyclus* genera have become models for identifying genetic mechanisms that generate phenotypic diversity and convergence [13–15]. Finally, the mechanisms of structural colour production via wing scale micro- and nanostructures have inspired technological innovations such as radiative cooling [16] and devices that detect ethanol and methanol vapours [17]. While a small number of butterfly taxa (e.g. *Ithominae, Heliconius, Morpho*, and *Bicyclus*) have provided detailed and mechanistic information on butterfly colouration, there remains an incredible variety of biological phenomena that broader butterfly diversity offers. Here we leverage the diversity of Australian butterflies by creating a comprehensive butterfly database that captures phenotypic variation. This open-access resource represents around a quarter of Australian butterfly diversity [18] and offers a valuable tool for assessing intraspecific variation in appearance and morphology. Key features include highly standardized UV and RGB images, along with spectral data, all of which facilitate an objective analysis of colouration. Genetic information (CO1) is linked to individual phenotypes. These data are available for download via Zenodo (full dataset) or the R package *ButtR*, which enables users to select, download, and install a subset of images. Overall, the database presents a novel and versatile data source for butterfly-related studies.

Australian butterfly fauna contains over 400 species [18], with many species still being discovered [19–21]. Australia is uniquely positioned because the wide-spread arid areas in the centre of the continent are inhospitable environments for butterflies, which reduces species richness despite the continent’s large geographic area [22,23]. Yet the east coast of Australia has undergone several events of adaptive radiation, where morphologically distinct species are characterised by low genetic diversity [24,25]. Indeed, Gross et al. [26] found that the Australian butterfly fauna together with the Sunda Shelf, represents a distinct phyloregion when compared to other terrestrial taxa. A key feature of butterfly speciation is wing colour pattern [27], which is precisely what we capture in our dataset, with images of over 4 000 individuals representing more than 100 species and matching CO1 genetic data for 1 635 of these individuals. A traditional evolutionary prediction is that animals are more colourful at lower latitudes [28], but so far Australian butterfly fauna shows a contradicting pattern by being equally colourful at all latitudes [28]. These unexpected results increase the interest in the unique evolutionary patterns of Australian butterfly fauna. More community studies are necessary to understand the evolution of colour pattern and diversification of Australian butterflies. This avenue of research is urgent as more than 5% of known Australian butterflies are at high risk of extinction within the next 15 years [29]. We hope to contribute to this research by providing a snapshot in time of multiple communities to be explored by many fields of research.

This newly created database is important because images are a useful tool for investigating broad scale evolutionary patterns in animals and plants [28, 30,31]. While field guides and museum collections play an important role as the primary sources of such images, these are often captured under non-standardised conditions. Field guides provide information of taxonomic and geographic distribution but are often limited to use in specific geographic regions (e.g. Australia, Asia, Indo-Pacific) and generally do not provide quantitative information of intraspecific variation. Natural museum collections provide large numbers of samples encompassing morphological variation, but samples are often: 1) haphazardly collected, 2) subject to differences in preparation and storage, 3) scattered geographically and displayed by taxon, making it difficult to extract community data. Many museums have begun to digitise their collections, which frequently involves photographing whole displays and consequently reducing image resolution. Museum images often capture only one side of the animal (typically the dorsal view) and lack UV lighting, despite the widespread occurrence and importance of UV-reflective patches on butterfly wings [32–38]. Additionally, they often omit scale bars and colour standards, limiting their utility for detailed open access analyses. As a result, digitised museum specimens may have limitations (i.e. standardized lighting) for advanced image analysis techniques [31,39].

Our newly created OzButterflies database contains 8 772 images of 4 161 individual butterflies (1 599 females and 2 562 males) from 125 species and 71 genera representing five families. These were collected from 19 sites spanning 2 500 km of latitude, designed to encompass the wet tropical and temperate regions along the east coast of Australia. Specimens from the same locality and year represent the community of butterflies of that site and time. Butterflies were photographed using RGB (red, green, and blue) and UV (ultraviolet) filters. The aim of this database is to provide image data in the rawest possible format, preserving user flexibility in post-processing and analysis. By avoiding pre-calibrated outputs, the dataset allows researchers to apply processing pipelines best suited to their specific analytical need. To aid with calibration, all images contain two full-spectrum grey standards and are raw images, suitable for a multitude of image analyses such as quantitative colour pattern analyses [40] or the modelling of colour perception [41]. We also provide spectral data for multiple wing locations on up to three individuals of each species.

## Methods

**Figure 1:**
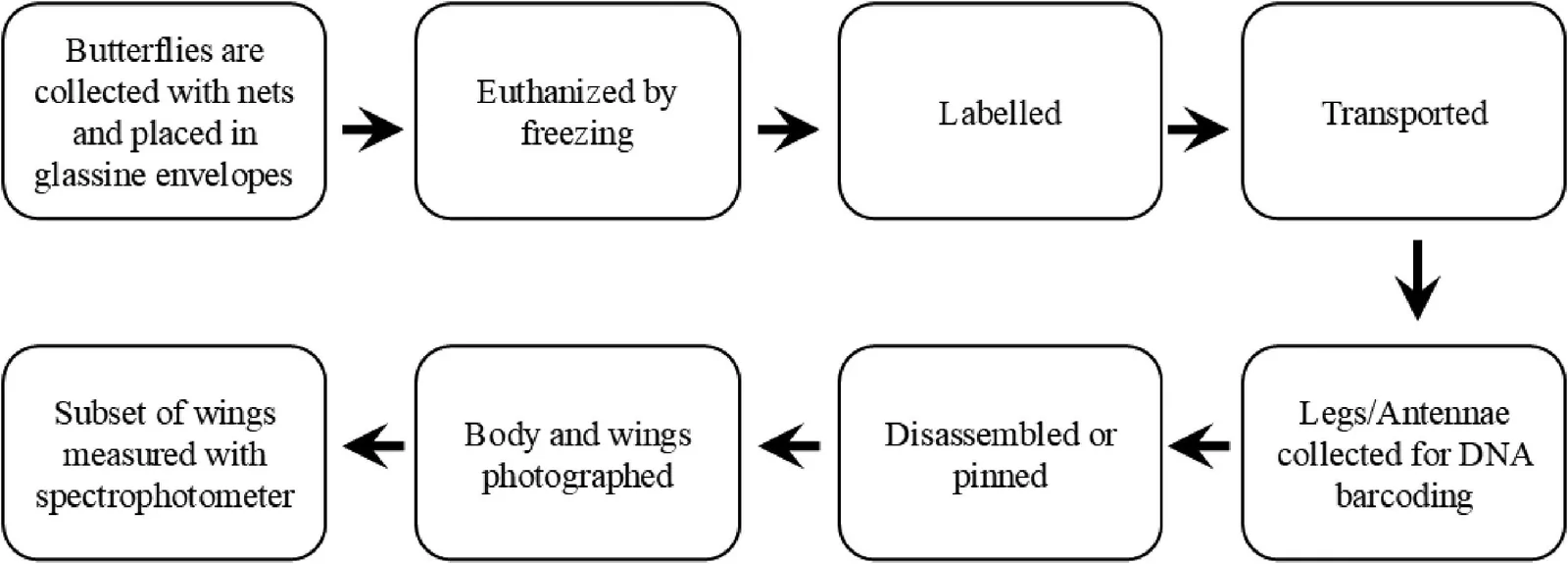
Sample collection and processing workflow. Butterflies were captured in the field and immediately placed in labelled envelopes inside a cooler bag to prevent dehydration and to anesthetize them. Freezing does not generally damage butterfly wings that are coloured by pigments but can damage structural colours if condensation occurs [42]. We minimized the effect of cooling by keeping the thawing cycles at a minimum, and by making sure the envelopes were dry. We removed at least one leg (when possible) or one antenna from a subset of individuals (*n* = 1851) for barcoding. Next, butterflies were either disassembled (n = 3936) or pinned (n = 225). Disassembled specimens were photographed immediately after disassembly, whereas pinned specimens were dried for a minimum of 48 h prior to photography. Finally, we used spectrophotometry to measure the reflectance of wing colour patches for representatives of each species.

We sampled free-flying adults from butterfly communities in tropical, subtropical, and temperate regions of eastern Australia over a period of two years (Table 1). Each site was sampled for 20 h within a week to represent a butterfly community. Butterflies were collected using insect nets (mesh size: 0.9 × 0.3 mm; hoop diameter: 456 mm) between 9:00 and 16:00 following the workflow described by Fig. 1. Four tropical and subtropical sites were sampled twice, once in in 2022 and once in 2023, whereas four temperate sites were sampled in 2022 and an additional four sites in 2023, totalling 480h of collection time across all sites and years. In each of the climate zones, we collected in two urban and two semi-natural habitats, always near a source of freshwater in order to maximize the chance of encountering butterflies as they are more commonly found near water. This site classification was visually assigned based upon vegetation density and the number of human visitors, with urban sites having lower density vegetation and higher human traffic than seminatural sites. Additionally, 27 specimens were provided to us as *ad hoc* donations (these can be identified as belonging to sites CCF, LG, or PD). We provide the GPS coordinates of the sites in the metadata (OzButterflies spreadsheet). The weather was mostly sunny on collection days; however, some collecting took place during light rainfall throughout the day.

**Table 1:** Summary of sites where samples were collected.

Site	Code	Years	Climate	Traditional owners
Cairns Botanic Gardens	BG	2022, 2023	Tropical	Gimuy-Walubarra Yidi
James Cook University	JCU	2022, 2023	Tropical	Djabugay, Yirrganydji, and Gimuy Yidinji
Gamboora Park	GP	2022, 2023	Tropical	Yidinji
Mossman River	MR	2022, 2023	Tropical	Kuku Yalanji
Brisbane Botanic Gardens Mt Coot-tha	BBG	2022, 2023	Subtropical	Turrbal and Jagera
Cabbage Creek	CC	2022, 2023	Subtropical	Turrbal
Oxley Creek	OC	2022, 2023	Subtropical	Yugara Ugarabul and Turrbal
Lakeside Park	LSP	2022, 2023	Subtropical	Jinibara, Kabi Kabi, and Turrba
Maquarie University	MQ	2022	Temperate	Wallumattagal clan of the Darug nation
West Pymple Park	WPP	2022	Temperate	Cammeraigal clan of the Kuringgai people
Ku-ring-gai Gardens	KRG	2022	Temperate	Darramurragal and Garigal
Westleigh Park	WLP	2022	Temperate	Darug and Guringai
Allan Small Oval	AO	2023	Temperate	Darug
Jubes Mountain Bike Track	JB	2023	Temperate	Darug
Woo-La-Ra Park	WLR	2023	Temperate	Gadigal clan
Parramatta Park	PP	2023	Temperate	Burramattagal people of the Dharug nation
Cabbage Creek Forest	CCF	2023	Subtropical	Turrbal
Lisgar Gardens	LG	2022	Temperate	Dharug and Guringai
Port Douglas City	PD	2023	Tropical	Yirrganydji

*Notes*: Code matches information on metadata. All sites were sampled in a standardised way for 20 h in total, with the exception of Cabbage Creek Forest, Lisgar Garden, and Port Douglas City where we took samples ad hoc.

### Photography

**Figure 2:**
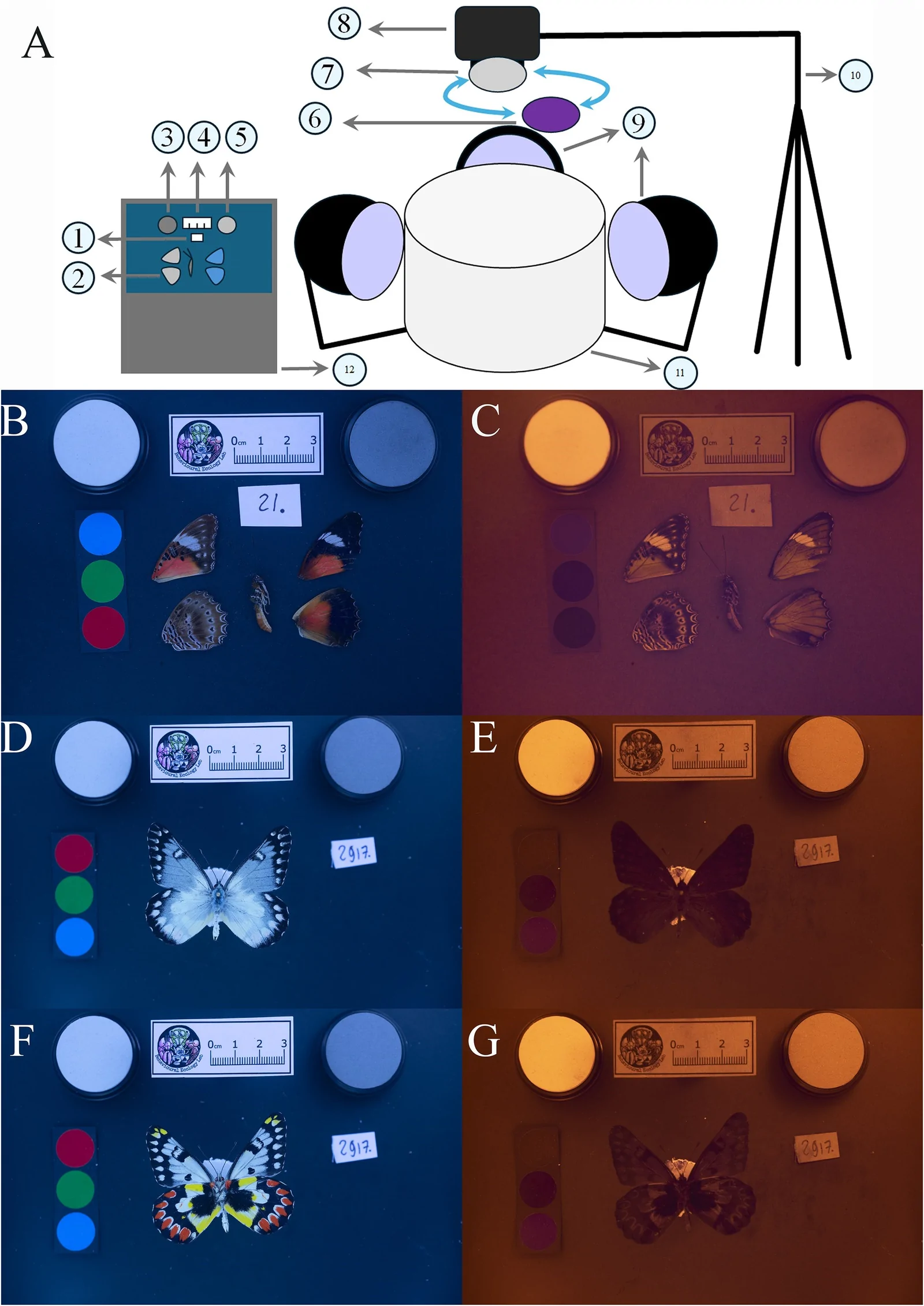
Photographic setup and sample images from the database. (A) 1. Specimen ID tag, 2. Specimen, 3. 60% full-spectrum grey standard, 4. Scale, 5. 20% full-spectrum grey standard, 6. UV filter, 7. Visible filter, 8. Camera, 9. UV lamps. 10. Tripod, 11. PTFE diffuser, 12. Computer. Samples were placed in the middle of the diffuser, and lamps were all positioned below the top of the diffuser at equal distances from each other. The camera was placed perpendicular to the specimen, supported by a C-stand. Photographs were taken using the remote shooter function on Imaging Edge software to minimize shaking. Filters were attached to the camera using a 3D printed filter holder and filters were swapped for RGB and UV images. B–G: examples of database images. (B) Disassembled RGB. (C) Disassembled UV. (D) Pinned dorsal RGB. (E) Pinned dorsal UV. (F) Pinned ventral RGB. (G) Pinned ventral UV.

We captured images using a Sony A7 camera body attached by bellows to an El Nikkor 80 mm lens. We converted the camera to a full spectrum configuration by removing the internal ‘hot mirror’ (UV/IR blocking filter) and replacing it with a clear optical window. Standard silicon sensors are intrinsically sensitive across the near-UV, visible, and near-IR range, and the factory hot mirror mainly restricts this range for conventional photography. Our conversion therefore did not extend the sensor’s fundamental sensitivity so much as remove this optical restriction, enabling broadband capture. We then defined the effective capture band using lens-mounted filters, as described below. This type of full-spectrum conversion is now common for mirrorless cameras and can be achieved via commercial modification services or equivalent specialist workflows. The camera was set to ISO 200, f/5.6, and a shutter speed of 1/15 s for RGB photos and 13 s for UV photos. Photographs were focused manually using the camera’s live view, with the RGB image used to achieve focus for each butterfly. For the RGB photo (human visible light) we used a *Baader UV-IR Cut* filter (420–685 nm) attached to the lens via a 3D printed filter swiper. This allowed us to switch rapidly between filters without disturbing the camera. For UV-only images, we used a *Baader Venus UV* filter (320–380 nm). All pictures included two full-spectrum grey standards (20% and 60% reflectance, Spectralon), with additional red, blue, and green colour standards of known reflectance (reflectance spectra for the standards are included in the database). The background of each image consisted of black cardboard. For pinned specimens, a small piece of white Styrofoam was also used to secure the specimen in position, and this piece may be visible in some images. Non-pinned images were imaged against a black background without Styrofoam. The light source used was an Exo Terra intense basking spot 75 W (UVA). We used three lamps diffused by a 1.5 mm thick PTFE ring (Swift Supplies) to reduce shadows. The sample was positioned flat on the Table, and the camera was mounted directly above it (perpendicular to the sample surface). Light sources were arranged symmetrically around the sample, slightly elevated above the plane of the specimen and angled downward toward it through the diffuser ring. For most specimens (n = 3937) we disassembled the wings from the body using sharp forceps so that the wings rested flat against the background, then took two images: one RGB and one UV. For these specimens the left wing represents the ventral colouration and the right wing represents the dorsal colouration. Some specimens remained intact (n = 225) as they were prepared (pinned) for museum vouchers. These individuals were selected based on visual inspection, prioritizing the best-preserved samples available at the time of imaging. Pinned specimens had four images taken: RGB and UV, both dorsal and ventral (Fig. 2). Photos were originally taken in .ARW format; however, because this format is not open-source, the files were converted to .DNG format in version 4 of the database. For users interested in the original .ARW files, these remain available in version 3 of the database on Zenodo. The associated R package allows users to select which database version to download, enabling them to obtain either the .DNG or the original .ARW files.

### Spectra acquisition

**Figure 3:**
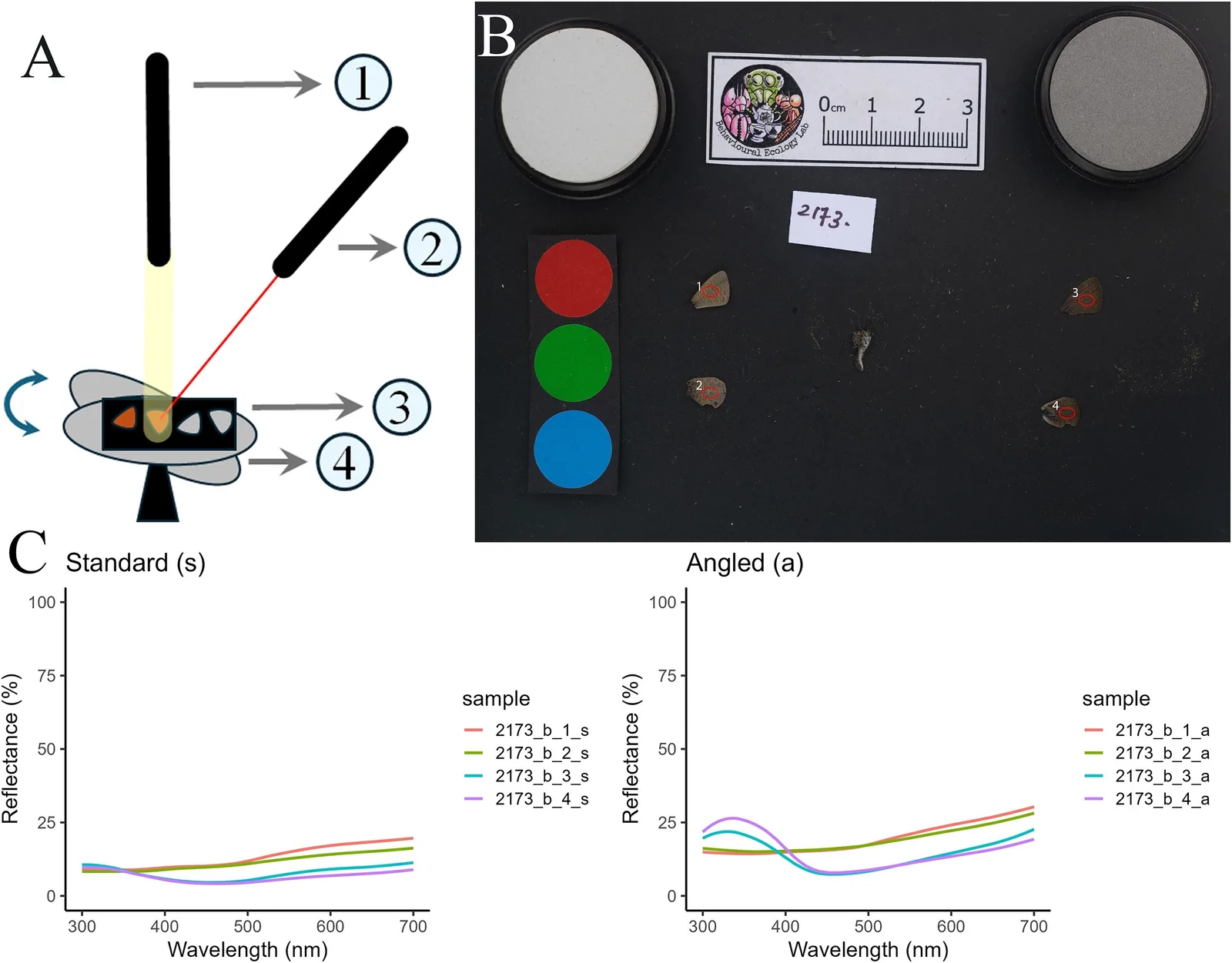
Representation of the spectroscopy workflow. (A) Spectrophotometer setup. 1. Lamp output was set perpendicular to the sample, 2. Laser guided optic fiber probe was set at 45^o^ to the sample, 3. Butterfly wings were taped flat onto black cardboard, 4. Turnstage was flipped 15^o^ to the right to obtain angled measurements. (B) Sample labels: red circles indicate the position on the wing that was measured by spectroscopy, and the numbers near the circles correspond to the spectra files and curves in sample graphs. (C) Sample spectral graphs: left spectrum was measured at 45^o^ and right spectrum at ~60^o^. For *Hypolimnas bolina*, iridescence was not detected with a 15° change in angle. Therefore, the wings were rotated 90° to capture the iridescence, and these measurements were labelled a2.

We selected the least damaged morphotype from each species for spectral measurement. This was based upon the number and size of wing tears and informed by a wing wear assessment (on a graded scale from 0 to 5) that accounted for the fading of colours and wing tip feathering [43]. We used a USB4000 spectrometer connected to a 1 mm Ø fibre-optic and a PX-2 xenon light source (all from Ocean Optics, Dunedin, Florida, USA). Spectral measurements were acquired with an integration time of 100 ms, a boxcar width of 10, and 20 scans averaged per measurement using SpectraSuite software (Ocean Optics). Probe size was set to ~2mm diameter. To ensure an accurate representation of wing colours, we visually selected wing patches based on the RGB and UV photograph of each sample. A measurement was taken for every patch which represented a visually distinct hue occupying a diameter of ~2 mm or greater. All wings were measured with optical fibre positioned at two different angles (45^o^ and 60^o^-75^o^) to assess the presence of iridescence (Fig. 3). Samples were labelled as iridescent when there was a visually apparent change in shape (peaks) between measures. The standard angle for non-iridescent patches was 45^o^, and we recommend that users use the 45° measurements unless specifically working on iridescence. All spectroscopic measurements are included in the OzButterflies database (as both ProSpec and CSV files). CSV files were generated by importing .procspec files into R using the pavo package [44]. Spectra were imported using the getspec() function (ext = ‘procspec’, decimal = ‘,’, lim = c(300, 700)). Negative values were then corrected, and the spectra were smoothed using the procspec() function (opt = ‘smooth’, fixneg = ‘addmin’, span = 0.25, bins = 5). Each butterfly that was measured has an additional image showing the locations of spectroscopic measurements. A Spectralon® 99% reflectance standard was used to calibrate the spectrometer between each sample.

### Sample identification

**Figure 4:**
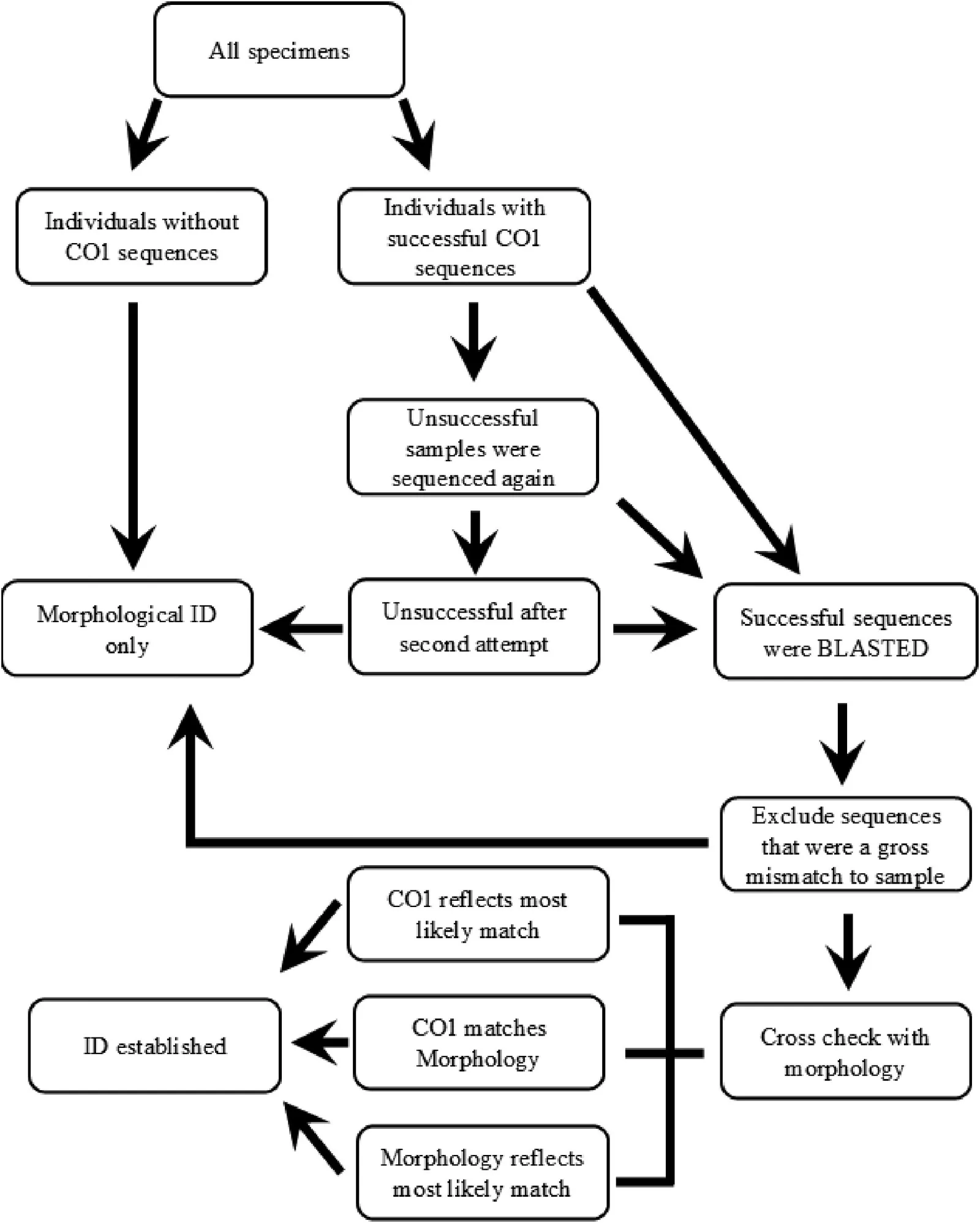
Flowchart of the processes used to ID specimens in the database. Specimens that did not have successful CO1 sequence amplification during PCR were identified on morphological features only, taking into consideration other species occurring in the community as determined by other CO1 sequences. Unsuccessful sequences that did not amplify during PCR or that had a high base pair uncertainty were re-sequenced (n = 44). Gross BLAST mismatches included cases where the sequence matched a different lepidopteran family or even a different Phylum (e.g. bacteria or fish). The remaining sequences were cross-validated against morphological ID. For most specimens, morphological and CO1 ID matched (n = 1523). COI match was considered the most likely match when the indicated species were visually similar to the specimen and occurred within Australia. Morphology was considered the most likely match when the CO1 match was not visually similar to the specimen, or when matched species did not occur within Australia.

Genomic DNA for molecular identification was extracted from legs and/or antennae of butterflies using prepGEM Universal (MicroGEM, Southampton, UK), a single-tube enzymatic extraction system following the manufacturer’s protocol. Legs and antennae were stored in 0.2 ml microfuge tubes at −30°C prior to DNA extraction. For each sample, 44.5 µL of UltraPure DNase/RNase-free distilled water (Invitrogen™), 0.5 µL prepGEM enzyme and 5 µL prepGEM 10X blue buffer were added with a total reaction volume of 50 µL. Samples were incubated at 75°C for 15 minutes and the enzyme denatured at 95°C for 5 minutes. The supernatant containing the extracted gDNA was then transferred to 1.5 mL microfuge tubes or 96-well plate (Eppendorf SE, Germany) and stored at −30 °C until PCR screening. Amplification of the CO1 gene was performed using previous published primers [45] LCO1490 (5′-ggtcaacaaatcataaagatattgg-3′) and HCO2198 (5′-taaacttcagggtgaccaaaaaatca-3′). Reactions comprised GoTaq Green Master Mix (Promega, Madison, USA), primers (0.4 µM), and 2.5 µL of extracted DNA in final reaction volume of 25 µL. Cycling conditions comprised an initial denaturation of 3 min at 94 °C followed by 40 cycles of 94 °C for 45 sec (denaturation), 45 °C for 45 sec (annealing), and 72 °C for 1 min (extension), with a final extension step at 72 °C for 5 min. Amplicons were purified for sequencing using ExoSAP-it and sequenced in the forward direction using LCO1490 at the Ramaciotti Centre, Sydney. Sequences were manually checked for read error and trimmed in Geneious (Dotmatics) [46]. A BlastN [47,48] was then performed using the Geneious NCBI BlastN tool to search NCBI nucleotide databases to identify closely matching sequences to each of the specimens in this study. To confirm specimen identification, we then checked the taxonomic information associated with the sequences in the GenBank database from the two closest % similarities identified through the BlastN [18]. Disagreements between morphology and CO1 sequence analysis were resolved by consensus between the lead authors (Fig. 4). Sequences are available in the BOLD system and the ‘Process ID’ relative to each deposited sequence can be found in the metadata spreadsheet. All samples collected in 2022 were submitted for sequencing; however, as sequencing was not successful for all individuals, species identification was confirmed using morphological criteria when necessary. The database includes 1635 successful CO1 sequences. Samples collected in 2023 were identified exclusively based on morphology. In total, 2 527 samples were identified solely using morphological characters.

## Technical validation

### Photography

**Figure 5:**
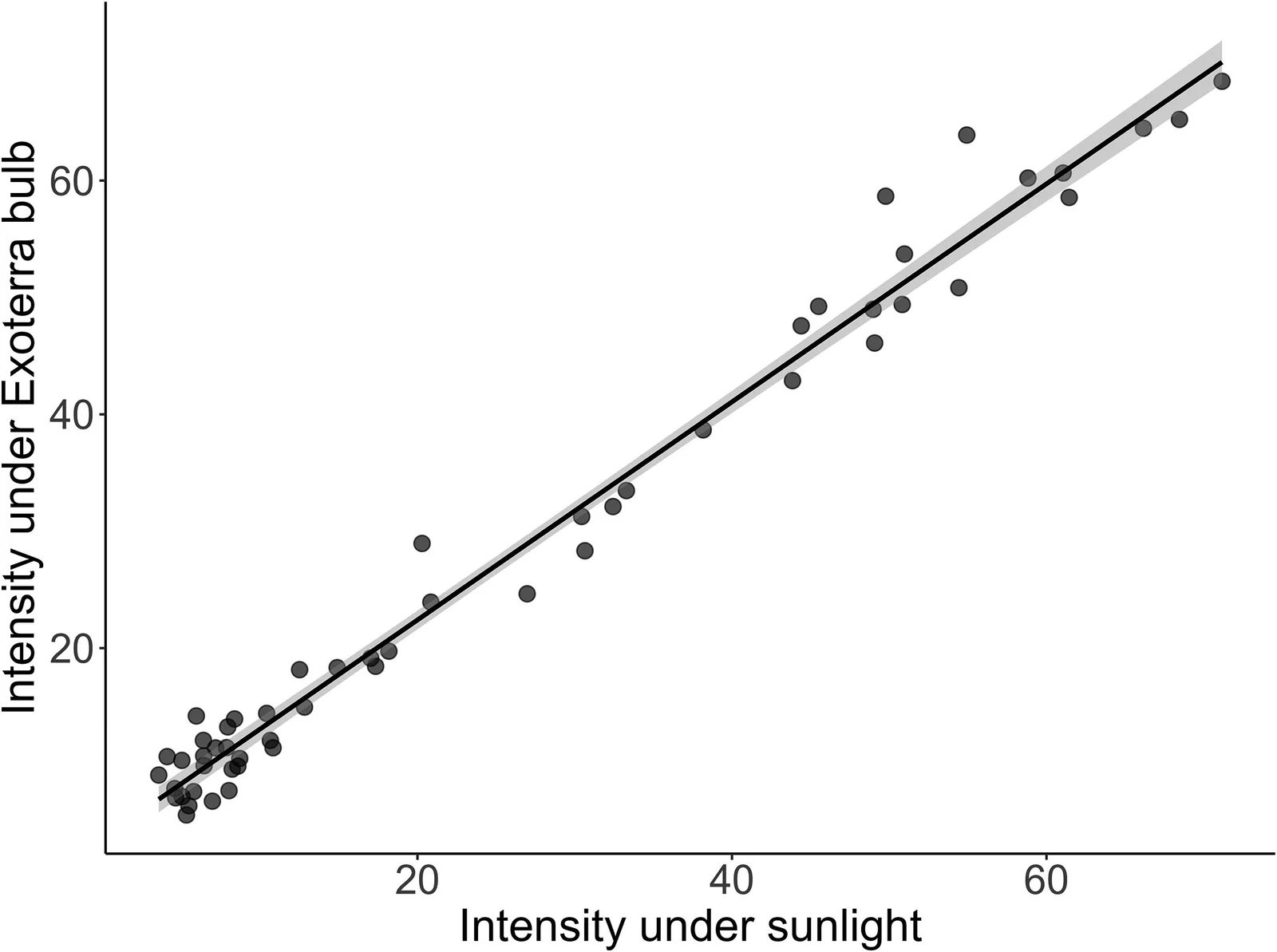
Correlation between wing brightness measured under sunlight and artificial illumination. Nine butterfly species were imaged under natural sunlight and under the artificial illumination (i.e. Exo Terra bulb). Multispectral image stacks were generated using MICA toolbox in ImageJ [49]. Identical colour patches were manually selected as regions of interest (ROIs) on aligned images for both illuminants. For each ROI, mean pixel intensity values were extracted for each channel (R, G, B, UV: b, UV: r) using the ‘Measure’ function in ImageJ. Brightness values obtained under the two illuminants were highly correlated (R² = 0.98).

All photographs were taken—and are stored in the database—in RAW format, which does not allow for post-processing. Consequently, images are not white-point calibrated and appear upside down. To validate the light source, we photographed a number of butterflies in both sunlight and illuminated with Exo Terra basking lamps, which contain UV light. RGB and UV values of the photographs in these different light conditions were highly correlated (R^2^ = 0.98, Fig. 5). We also visually confirmed that the butterflies of known UV patterns had the expected UV reflectivity in our setup. The Spectralon colour standards and scales also validate the photographs. To facilitate posterior colour analyses with existing software, we used a MICA toolbox [49] compatible camera (see methods), lenses, and filters to take the photographs. MICA Toolbox does not support .DNG files. Therefore, users interested in performing colour analyses with MICA should download version 3 of the database, which contains the original .ARW files. Furthermore, calibrated digital photography has been shown to yield biologically relevant visual-model outputs (including cone-catch–based measures) that agree closely with estimates derived from spectrometer reflectance when appropriate calibration is performed [49]. Users wishing to perform colour analyses from the photographs will need to calibrate the images themselves. Established calibration and analysis pipelines are available online (e.g. MICA toolbox [40, 49,50]: https://www.empiricalimaging.com/; pavo [44]: https://pavo.colrverse.com/).

### Spectroscopy

For the spectroscopy, we used a laser pointer to ensure that the optic fiber measured the correct patch of the butterfly wing. A laser pointer was aligned with the distal end of the optic fibre to indicate the precise measurement location. The projected laser spot corresponded to the area from which the probe collected reflectance, enabling accurate positioning on colour patches smaller than the diameter of the light source (~5 mm). After verifying the measurement position, the laser was turned off, and the fibre was reconnected to the spectrometer prior to reflectance acquisition. Spectra were visually checked to confirm that they matched the expected visible colour, and mismatching patches were remeasured. Spectra of iridescent patches were visually checked to confirm that reflectance spectra varied with the angle of measurement.

### Sample identification

Information between spreadsheets and photographs was cross-checked with an initial species ID acquired during collection to ensure that samples matched the original collection data. Morphological IDs were verified by more than one observer. DNA sequences always had a positive and negative control for PCR verification. The strength of bands was verified by running the PCR product on an agarose gel and visualising the gel prior to sending the samples for sequencing. Samples that were not of appropriate strength were discarded. DNA sequences that matched to non-lepidopteran taxa (e.g. bacteria), or that had a high number of uncertain base pairs were discarded (n = 83). When DNA and morphological ID disagreed, the lead authors discussed case by case the source of disagreement to reach a consensus on identification using additional online verified images. The identities of photographed butterflies were checked manually, with file names corrected as necessary. In cases where the sample ID in the photograph and the file name differ, the file name (and the name of the containing folder) gives the correct sample name.

## Results

**Figure 6:**
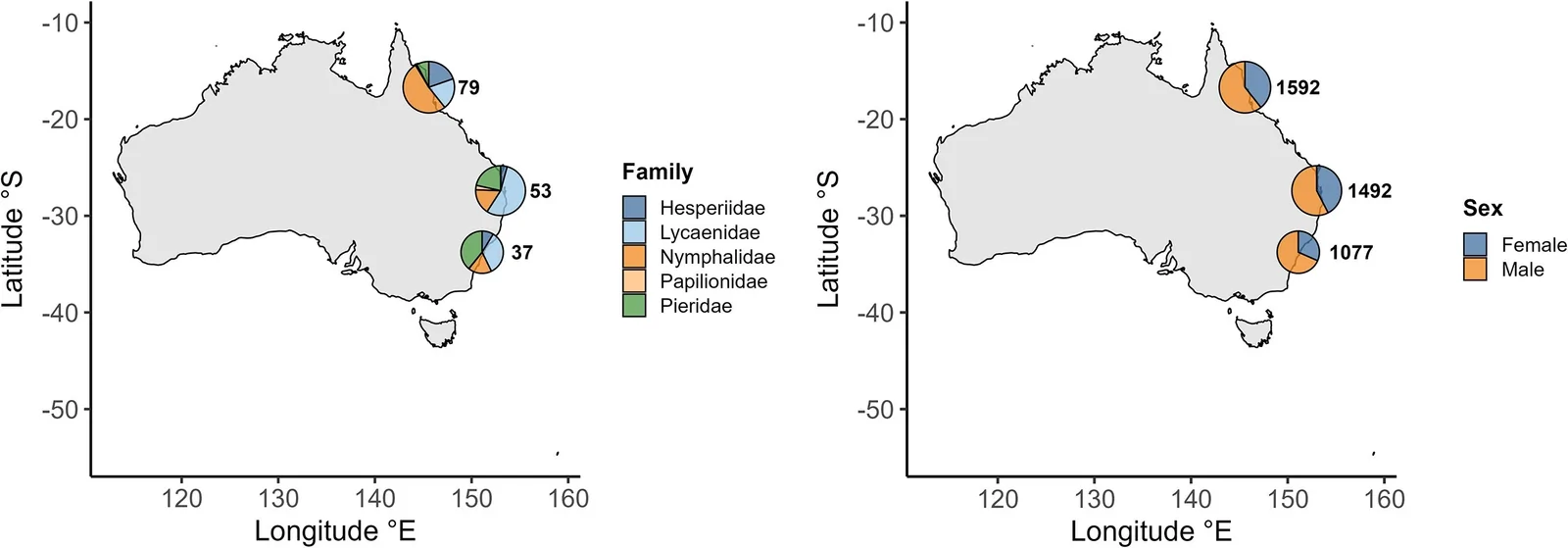
Summary of database samples. (A) Distribution of families collected in three climate zones (temperate, subtropical and tropical); numbers represent the total number of species for each zone. (B) Distribution of males and females collected in three climate zones (temperate, subtropical and tropical); numbers represent the number of samples per climate zone.

OzButterflies contains information from 4 161 individuals (1 599 females and 2 562 males) from 125 species (Table 2), collected at 19 sites along the east coast of Australia located near Cairns, Brisbane and Sydney (Fig. 6). The database is released under a CC0 1.0 license with unrestricted use. The database is available for download from Zenodo (10.5281/zenodo.15881960). Within Zenodo, the database is stored as descriptive metadata files with sample data in ZIP files (one ZIP file per species) and includes a README.txt file. When extracted, the ZIP files expand to data files within family, species and sample folders. In addition, we provide an R package, *ButtR*, that allows a subset of the database, as chosen by the user, to be downloaded, saving download time and disk space when the entire database is not required. *ButtR* can also be used to simplify download and install the entire database. *ButtR* can be installed from CRAN, and source code is available on GitHub (https://github.com/DiogoJackson/ButtR). Instructions for use are available at https://github.com/DiogoJackson/ButtR.

**Table 2:** List of species of Australian butterflies in the OzButterflies database.

Family	Genus	Species	Author
Hesperiidae	*Arrhenes*	*dschilus*	Mabille, 1904
	*Cephrenes*	*augiades*	Felder, 1860
	*Hesperilla*	*crypsigramma*	Meyrick & Lower, 1902
		*picta*	Leach, 1814
	*Mesodina*	*halyzia*	Hewitson, 1868
	*Netrocoryne*	*repanda*	C. Felder & R. Felder, 1867
	*Notocrypta*	*waigensis*	Plötz, 1882
	*Ocybadistes*	*ardea*	Bethune-Baker, 1906
		*flavovittatus*	
		*knightorum*	Lambkin & Donaldson, 1994
		*walkeri*	Heron, 1894
	*Parnara*	*amalia*	Semper, 1879
	*Pelopidas*	*agna*	Moore
		*lyelli*	Rothschild, 1915
	*Sabera*	*caesina*	Hewitson, 1866
		*dobboe*	Plötz, 1885
		*fuliginosa*	Miskin, 1889
	*Suniana*	*lascivia*	Rosenstock, 1885
		*sunias*	Felder, 1860
	*Tagiades*	*japetus*	Stoll, 1781
	*Taractrocera*	*dolon*	Plötz, 1884
	*Telicota*	*ancilla*	Herrich-Schäffer, 1869
		*anisodesma*	Lower, 1911
		*augias*	Linnaeus, 1763
		*colon*	Fabricius, 1775
		*mesoptis*	Lower, 1911
		*ohara*	Plötz, 1883
	*Toxidia*	*peron*	Latreille, 1824
	*Trapezites*	*praxedes*	Plötz, 1884
		*symmomus*	Hübner, 1923
Lycaenidae	*Arhopala*	*micale*	Blanchard, 1853
		*wildei*	Miskin, 1891
	*Candalides*	*absimilis*	Felder, 1862
		*cyprotus*	Olliff, 1886
		*erinus*	Fabricius, 1775
		*hyacinthinus*	
		*margarita*	Felder, 1860
	*Catochrysops*	*panormus*	Felder, 1860
	*Catopyrops*	*ancyra*	Felder, 1860
		*florinda*	Butler, 1877
	*Deudorix*	*diovis*	Hewitson, 1863
	*Erysichton*	*lineatus*	
	*Euchrysops*	*cnejus*	Fabricius, 1798
	*Famegana*	*alsulus*	Herrich-Schäffer, 1869
	*Hypochrysops*	*polycletus*	Linnaeus, 1758
		*pythias*	C. Felder & R. Felder, 1865
	*Hypolycaena*	*phorbas*	Fabricius, 1793
	*Jamides*	*aleuas*	C. Felder & R. Felder, 1865
		*bochus*	Stoll, 1782
		*phaseli*	Mathew, 1889
	*Lampides*	*boeticus*	Linnaeus, 1767
	*Leptotes*	*plinius*	Fabricius, 1793
	*Megisba*	*strongyle*	Felder, 1860
	*Nacaduba*	*berenice*	Herrich-Schäffer, 1869
		*biocellata*	C. Felder & R. Felder, 1865
		*cyanea*	Cramer, 1775
		*kurava*	Moore
	*Neolucia*	*mathewi*	Miskin, 1890
	*Paralucia*	*aurifera*	
	*Prosotas*	*dubiosa*	Semper, 1879
		*felderi*	Murray, 1874
	*Psychonotis*	*caelius*	Felder, 1860
	*Theclinesthes*	*onycha*	Hewitson, 1865
		*sulpitius*	Miskin, 1890
	*Zizina*	*otis*	Fabricius, 1787
	*Zizula*	*hylax*	Fabricius, 1775
Nymphalidae	*Acraea*	*andromacha*	Fabricius, 1775
		*terpsicore*	Linnaeus, 1758
	*Cethosia*	*cydippe*	Linnaeus, 1767
	*Cethosia*	*penthesilea*	Cramer, 1777
	*Cupha*	*prosope*	Fabricius, 1775
	*Danaus*	*affinis*	Fabricius, 1775
		*petilia*	Stoll, 1790
		*plexippus*	Linnaeus, 1758
	*Doleschallia*	*bisaltide*	Cramer, 1777
	*Euploea*	*corinna*	
		*darchia*	MacLeay, 1826
		*sylvester*	Fabricius, 1793
		*tulliolus*	Fabricius, 1793
	*Heteronympha*	*merope*	Fabricius, 1775
		*mirifica*	Butler, 1866
	*Hypocysta*	*irius*	Fabricius, 1775
		*metirius*	Butler, 1875
	*Hypolimnas*	*alimena*	Linnaeus, 1758
		*bolina*	Linnaeus, 1758
	*Junonia*	*hedonia*	Linnaeus, 1764
		*orithya*	Linnaeus, 1758
		*villida*	Fabricius, 1787
	*Melanitis*	*leda*	Linnaeus, 1758
	*Mycalesis*	*perseus*	Fabricius, 1775
		*sirius*	Fabricius, 1775
		*terminus*	Fabricius, 1775
	*Mynes*	*geoffroyi*	Guérin-Méneville
	*Neptis*	*praslini*	Boisduval, 1832
	*Pantoporia*	*consimilis*	Boisduval, 1832
	*Phaedyma*	*shepherdi*	Moore, 1858
	*Tirumala*	*hamata*	MacLeay, 1826
	*Tisiphone*	*abeona*	Donovan, 1805
	*Vagrans*	*egista*	Cramer, 1780
	*Vanessa*	*itea*	Fabricius, 1775
		*kershawi*	McCoy, 1868
	*Yoma*	*sabina*	Cramer, 1780
	*Ypthima*	*arctous*	Fabricius, 1775
Papilionidae	*Cressida*	*cressida*	Fabricius, 1775
	*Graphium*	*agamemnon*	Linnaeus, 1758
		*choredon*	
	*Pachliopta*	*polydorus*	Linnaeus, 1763
	*Papilio*	*aegeus*	Donovan, 1805
		*ambrax*	Boisduval, 1832
		*demoleus*	Linnaeus, 1758
Pieridae	*Belenois*	*java*	Sparrman, 1768
	*Catopsilia*	*pomona*	Fabricius, 1775
	*Cepora*	*perimale*	Donovan, 1805
		*aganippe*	Donovan, 1805
		*argenthona*	Fabricius, 1793
		*mysis*	Fabricius, 1775
		*nigrina*	Fabricius, 1775
	*Elodina*	*angulipennis*	Lucas, 1852
		*parthia*	Hewitson, 1853
		*queenslandica*	De Baar & Hancock, 1993
	*Eurema*	*brigitta*	Stoll, 1780
		*hecabe*	Linnaeus, 1758
		*laeta*	Boisduval, 1836
		*smilax*	Donovan, 1805
	*Pieris*	*rapae*	Linnaeus, 1758

*Notes*: All names according to Braby (2016).

When installed, the database consists of multiple files organised within a folder hierarchy. The top-level folder contains a README.txt file that describes the database contents, two spreadsheets and reflectance spectra for the colour standards (.ProcSpec format). The spreadsheets are a descriptive database summary (‘Oz_butterflies_summary’) and provide detailed information about each specimen in the database (‘Oz_butterflies’). Both spreadsheets are provided in CSV, Microsoft Excel (.xlsx) and JSON formats. The summary spreadsheet includes the number of individuals, and the number of males and females for each species, as well as some other basic information such as whether the species is sexually dimorphic or iridescent. Sexual dimorphism was considered when there was a clear morphological difference between male and female morphotypes (e.g. *Hypolimnas bolina* is sexually dimorphic, *Arrhenes dschilus* is not dimorphic). Iridescence was considered present when we saw changes in the reflectance of specimens when measuring the spectra at different angles coupled with visual information when tilting specimens. The specimen spreadsheet contains each specimen’s ID, the best available species ID, where, when and by whom it was collected, and if there is damage on the wings or body (we did not classify brushed-off scales as damage; small wing tears on the wing edge were considered damage, while tears within the wing were not).

In addition to the spreadsheets, the database contains photographs, spectroscopic output files and CO1 sequence data. The top-level folder contains a folder for each butterfly family. Family folders contain a folder for each species, and species folders contain a folder for each specimen, named with the specimen ID. At a minimum, each specimen has RGB and UV photographs. Some samples (at least one per species) include spectroscope output files and/or CO1 barcode files. Photographs are in Sony raw format (.dng). Spectroscope output files are in ProcSpec and CSV formats. CO1 barcode files are in ab1 (.ab1) format.

## Code availability

The source code for the *ButtR* R package is publicly available on GitHub at https://github.com/DiogoJackson/ButtR. The GitHub repository also includes the code used to package the database content in preparation for uploading to Zenodo.

## Discussion

The OzButterflies database can be used to explore phenotypic variation within and between butterfly communities. It has the potential to inform multiple current hypotheses regarding phenotypic variability, such as sexual dimorphism, climate-related expression of colouration, and the distribution of conspicuous colours across families and regions. The associated spectral measurements should allow exploration of a multitude of topics, including prey defensive strategies, sexually selected colouration, and patterns of wing melanisation. Given that all photographs include a scale, it is also possible to extract measures of morphometric traits, such as size and width of body and wings, to estimate parameters such as flight capabilities, or symmetry in wing shape. Community information could be combined with meteorological or environmental databases to map and explore relationships between environment, colour and size expression/variation. We also present new basic information for some species such as the presence of iridescence and sexual dimorphism in ultraviolet wavelengths. Spectra and photographs can be modelled according to the visual systems of different animals to estimate how predators and conspecifics see butterfly colouration. The OzButterflies database thus represents a vast and highly versatile tool for addressing a variety of ecological and evolutionary questions within and beyond the field of lepidopteran biology and is certainly a resource that several of our authors wished they had when they started their academic research.
